# Efficacy of an inactivated and adjuvanted “ZULVAC^®^ 8 OVIS” vaccine produced using single-use bioreactors

**DOI:** 10.1186/1753-6561-5-S1-P118

**Published:** 2011-11-22

**Authors:** Lídia Garcia, Helena Paradell, Mercedes Mouriño, Berta Alberca, Alicia Urniza, Ana Vila, Margarita Tarrats, Joan Plana-Durán

**Affiliations:** 1 Pfizer Olot S.L.U., Ctra. Camprodon s/n, La Riba, 17813 Vall de Bianya (Girona), Spain

## Introduction

Bluetongue virus (BTV) first emerged in the European Union (EU) in 2006, peaking at 45,000 cases in 2008. The EU spent million of euros in 2008 and 2009 on eradicating and monitoring programs, co-financed with member states. The number of cases in 2009 was 1118, with only 120 reported across the EU so far last year. Vaccination has proven itself as the most effective tool to control and prevent the disease and to facilitate the safe trade of live animals.

Mammalian cells are commonly used as a substrate for production of most of the viral vaccines. BHK-21 cells are used for the production of bluetongue vaccines. Most companies use roller bottles or conventional bioreactors, but taking into account that the manufacturing cost is very important, the possibility of using Single-Use Bioreactor (SUB) technology as an alternative to roller bottles or conventional bioreactors was explored. Advantages like yields of production, time reduction (elimination of cleaning and sterilization steps needed for bioreactors, no validation process, etc) and quality of antigen production were studied.

## Materials and methods

### Cell line

*BHK-21* cell line was used due to the good yields. Cells were cultured at 37°C in Minimum Essential Medium Glasgow supplemented with serum.

### Cultivation system

The growth of the BHK-21 cells and virus production was conducted in roller bottles (RB), 10-L bioreactor (Biostat B-plus) and in 250-L SUB (Hyclone).

Cells on bioreactors and SUB were cultivated using microcarriers (Cytodex-3) at a density of 3g/L. The crystal violet dye nucleus staining method was used to estimate cell density.

Dissolved oxygen (DO) and pH were automatically adjusted by addition gases (CO_2_, and air).

### Virus strain

BTV serotype 8 (BTV-8), strain BEL2006/02 was used in all experiments. The strain was supplied by “Veterinary and Agrochemical Research Centre” (VAR-CODA-CERVA), Ukkel, Belgium.

Once the cells were 80-100% confluent, RB, 10-L bioreactor and 250-L SUB were infected under identical conditions and with a constant multiplicity of infection (MOI). Harvesting of virus was done when cytopathic effect (CPE) was about 90- 100%.

Virus production was evaluated by TCID_50_/ml.

### Vaccine

The antigen obtained on SUB was inactivated by Binary Ethylenimine (BEI) and adjuvanted with aluminum hydroxide and saponin. The efficacy of the vaccine was tested in lambs.

### Animals and experimental design

Forty, 1.5 month-old, lambs were included in the study.

Thirty lambs were vaccinated and revaccinated 3 weeks later by subcutaneous route and ten lambs were left as unvaccinated controls. Forty-four days after revaccination all lambs were challenged with BTV-8.

The antibody response in vaccinated lambs was evaluated from vaccination until the moment of challenge by a seroneutralization test (see table 1).

**Table 1:** NA titers against BTV serotype 8 after vaccination

	Mean NA Titers			
**Group**	**D<0**	**D+35**	**D+42**	**D+65**

vaccinated	<2	88,4	34,7	50,5
unvaccinated	<2	<2	<2	<2

D<0:before first vaccination

D+35: 2 weeks after revaccination

D+42: 3 weeks after revaccination

D+65:before the challenge

Viremia (presence of BTV genome in blood samples) was evaluated by real time qRT-PCR [1] in blood samples obtained before challenge and during four weeks after challenge.

## Results and discussion

Some experiments to scale-up were done. An optimized and transferable process was developed in 10L glass vessels by monitoring the pH, temperature, and DO. The final process was transferred to 250-L stirred-tank SUB integrated with an Applikon controller.

Cell growth and virus production in SUB was conducted at the optimal conditions determined previously on conventional bioreactors. Three critical parameters were taken into account: Cell concentration, growth rate at start virus inoculation and harvesting time.

### Cell growth

Several studies were conducted to compare the growth of cells in RB, 10-L bioreactor and 250-L SUB. During culture in bioreactors and SUB, DO was regulated by pulse of oxygen and pH was controlled.

Figure 2, shows that cell concentration at 48 hours (when confluency is reached) was higher in bioreactors than in roller bottles. That corresponds to an increase of cell biomass by a factor of 1.5.

**Figure 1:**
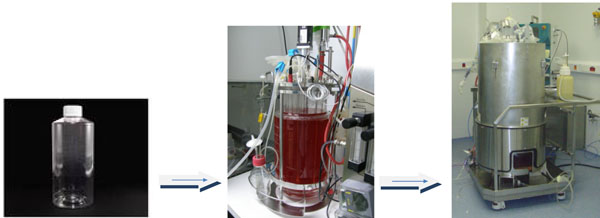
Scale-up from roller bottles to 250-L SUB

#### Virus production

Figure 3, shows the results of the virus titers in 250-L SUB compared with those obtained in RB and 10-L bioreactor.

**Figure 2:**
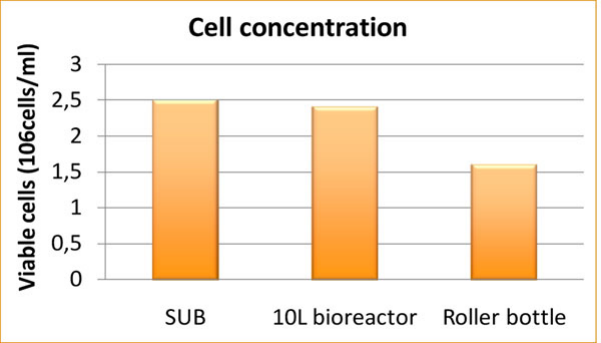
BHK-21 concentration at 48 hours post cultivation.

**Figure 3:**
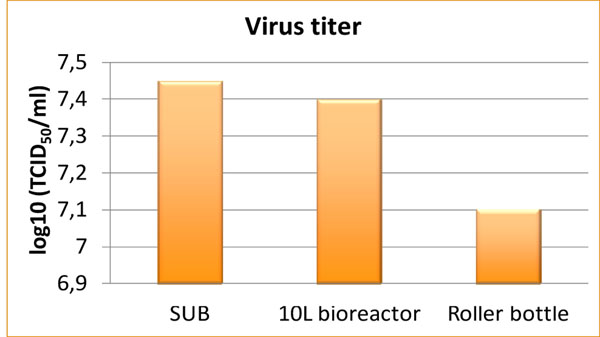
Virus titers obtained at 72h p.i.

The virus titer reached in a 10L bioreactor was comparable with the levels obtained in SUB. Preliminary results prove that by using SUB, the yields obtained in roller bottles can increase more than two times.

### Efficacy study

The potency of antigens produced using 250-L SUB technology was verified in target species.

#### Serological study

Determination of neutralizing antibodies (NA) titer against BTV serotype 8 in the samples taken after vaccination and revaccination was done by means of seroneutralization test. Table 1 show the geometric mean NA titers.

#### Evaluation of viremia after challenge

In any of the lambs of the vaccinated and challenged groups, viral gemone could be detected by RT-PCR during 27 days after challenge. Whereas in all unvaccinated and challenged lambs, the viral genome was detected from day 3-5 p.i.

## Conclusions

• The results obtained using a 250-L SUB concerning the cell concentration and virus titer, were similar to those reached in a 10L bioreactor and higher than in roller bottles.

• Efficacy results on lambs confirm that the quality of antigen produced in SUB are similar of the antigen produced both in roller bottles and on conventional bioreactor.

• We can think on the possibility of using disposable systems in vaccines production in order to reduce the production costs.

• SUB can be an alternative to conventional production methods:

⠼ Reduced facility complexity, reduction of the cost of building, and possibility of the rapid expansion of the capacity of the production.

⠼ Reduction of the capital of equipment and equipment validation, etc.

⠼ Avoid the cleaning process and reduction of the risk of cross-contamination.
